# Sociodemographic and clinical features of men and women with eating disorders: a diagnosis-matched, retrospective comparison among inpatients

**DOI:** 10.3389/fpsyt.2023.1192693

**Published:** 2023-07-07

**Authors:** Philipp Traut, Georg Halbeisen, Karsten Braks, Thomas J. Huber, Georgios Paslakis

**Affiliations:** ^1^University Clinic for Psychosomatic Medicine and Psychotherapy, Medical Faculty, Campus East-Westphalia, Ruhr-University Bochum, Luebbecke, Germany; ^2^Centre for Eating Disorders, Klinik am Korso, Bad Oeynhausen, Germany

**Keywords:** anorexia nervosa, bulimia nervosa, binge-eating disorder, gender differences, psychotherapy

## Abstract

**Introduction:**

Eating disorders (EDs) are among the most severe mental disorders in women and men, often associated with high symptom burden and significant limitations in daily functioning, frequent comorbidities, chronic course of illness, and even high mortality rates. At the same time, differences between men and women with EDs remain poorly explored.

**Methods:**

In this study, we compared 104 men to 104 diagnosis-matched women with EDs regarding sociodemographic and clinical features. Using latent class mixture modelling, we identified four distinct patient subgroups based on their sociodemographic features.

**Results:**

Men with EDs had significantly higher odds than women to belong to a “single-childfree-working” class. Moreover, while there were few overall differences in ED-related symptoms and general psychopathology between men and women, single-childfree-working men with EDs presented with higher general psychopathology symptoms than men in the other classes.

**Discussion:**

We discuss how considering sex and gender along with further sociodemographic differences in EDs may help to improve ED diagnosis and treatment.

## 1. Introduction

Eating disorders (EDs), characterized by disturbed eating patterns, body image concerns, and weight-control behaviors (1), are among the most severe mental disorders in terms of psychological burden, rates of chronification, and mortality (2–4). Contrasting stereotypical perceptions of a “women’s disease” (5, 6), EDs are increasingly recognized as a health risk in men. Indeed, prevalence and burden have risen faster in men than women over the last 30 years (7, 8). Global estimates suggest that up to 8.4% of women and 2.2% of men suffer from EDs such as Anorexia Nervosa (AN), Bulimia Nervosa (BN), or Binge-Eating Disorder (BED) during their lifetime (9–11). These estimates imply that men may constitute up to every fourth clinical ED case.

Men, however, remain underrepresented in ED research and care (12, 13), despite recent findings suggesting a considerable role of sex and gender^1^ for ED presentation in terms of disordered eating and general psychopathology (14–16). For example, men with AN often pursue reducing body fat to make muscles more visible (17–19). Men with BN show greater preference for high-protein and high-fat food during binge-eating episodes and consume objectively larger amounts of foods than women, while at the same time men with BN and BED report to experience less loss of control and psychological distress than women (20, 21). Studies using standardized ED assessments, such as the Eating Disorder Examination-Questionnaire [EDE-Q; (22)], found adolescent women with EDs to present with higher symptom scores relative to adolescent men with EDs (23), and there are similar patterns in community samples (24). However, the overall findings remain inconclusive, as other studies suggest gender parity in severity levels of ED-related and general psychopathology (25, 26), or even found that men with EDs were more likely than women to present with depressive symptoms (27) and other comorbidities such as psychosis and drug abuse (28).

Given the overall scarce and somewhat mixed results of gender comparisons in clinical ED populations, further comprehensive investigations on gender differences in disordered eating and general psychopathology are needed. In addition, only a few studies have explored sex and gender differences regarding sociodemographic factors of individuals with EDs, and these studies usually limited comparisons to single dimensions. For example, one study found that men presented to treatment at a younger age than women and were more often Nonwhite (23), whereas another study reported on the lack of age differences at admission to treatment (29). A review found stronger associations between self-reported sexual harassment and ED-related psychopathology in men than in women (30). Other examples include observations that ED-related symptomatology is shared by mothers and daughters but not by mothers and sons (31), and that low social support predicts disordered eating in boys but not in girls (32). However, we are unaware of studies using multidimensional (clustering) approaches for the detection of gender-associated sociodemographic patterns among patients with EDs, although these approaches could be particularly informative for identifying vulnerable groups and tailoring prevention and treatment strategies (33).

The present study sought to compare clinical and sociodemographic features between men and women with EDs. Using standard admission data from an ED specialty clinic, we evaluated and compared ED-related and general psychopathology between diagnosis-matched men and women with AN, BN, BED, and EDNOS (Eating Disorder Not Otherwise Specified). We pursued matched-samples rather than convenience-samples comparisons, as the prevalence of specific ED diagnoses differs between men and women (34), and women with EDs outnumber men with EDs in general and clinical populations (12). This usually results in unbalanced comparisons between men and women in convenience samples that render statistical inference unreliable, and in non-orthogonal data structures that make it difficult to distinguish between gender-based and diagnosis-based effects. By matching men with EDs to an identically sized sample of women with identical diagnosis, gender comparisons become balanced, and gender and diagnosis vary orthogonally, improving the reliability of statistical inference.

Going beyond previous research, we further identified patterns in sociodemographic features such as educational level, relationship status, and living circumstances using latent class analysis (35), a method for identifying distinct subgroups within populations that share observable features (36). We were particularly interested in (a) whether patient gender is associated with patterns of sociodemographic features, and (b) how sociodemographic patterns relate to ED and general psychopathology. Given the paucity of sex- and gender-based comparisons in samples of inpatients with EDs, our approach remained exploratory, and we refrained from making strong predictions about the magnitude and direction of effects.

## 2. Materials and methods

### 2.1. Participants

For this study, we selected and compared a subsample of individuals with EDs originally described elsewhere (15, 37). Specifically, we selected first admission datasets of 104 men with EDs (27 AN, 11 BN, 59 BED, 7 EDNOS) who completed both diagnostic and sociodemographic assessments and had been admitted and treated between January 2018 and December 2021 at the Klinik am Korso, Bad Oeynhausen, Germany. All 104 datasets of men were matched to 104 first admission datasets from women with EDs who completed diagnostic and epidemiological assessments (104 out of a total of 577 women with AN, 397 women with BN, 215 women with BED, and 29 women with EDNOS), who had been treated at the same clinic during the same time. In contrast to previous analyses that compared end-of-treatment outcomes for individuals matched according to at-admission characteristics (15), the quasi-random matching for the present study was based on diagnostic group only, allowing for meaningful comparisons of at-admission characteristics between gender and diagnostic groups.

All individuals with EDs were diagnosed according to ICD-10 criteria (38) based on the clinical judgment of long-term experts in the diagnosis and treatment of EDs (trained physicians and psychologists), and diagnoses were validated by means of peer review (therapists’ meetings). Binge-eating disorder diagnoses were classified under a category other than EDNOS; in other words, the EDNOS category had no BED diagnoses. Inpatient treatment followed a multimodal rehabilitation concept based on psychodynamic and cognitive-behavioral approaches and included individual and group psychotherapy, psychoeducation, nutritional rehabilitation, and complementary therapies (e.g., body psychotherapy). As of late March, 2020, contact restrictions enacted as part of the Covid-19 pandemic response required wearing masks and limiting inter-patient contact, but left the therapy program unaltered.

The retrospective analyses were reviewed and approved by the Ethics Committee of the Ruhr-University Bochum’s Medical Faculty at Campus East-Westphalia as part of application AZ 2021-849 and registered with the German Clinical Trial Register as part of application DRKS00028441. Datasets are available from the corresponding author upon reasonable request.

### 2.2. Assessments

Primary diagnoses, age, gender, body height, and body weight at admission (used to calculate the Body Mass Index, BMI) were extracted from the clinic’s records. Height was measured by the nursing team, body weight was assessed using a calibrated scale (KERN & SOHN GmbH, Balingen-Frommern, Germany). Extracted records additionally included standardized psychopathological and sociodemographic assessments, as detailed below.

#### 2.2.1. Eating disorder symptoms

Symptoms of EDs were assessed using the validated German translation of the EDE-Q (39), which includes 22 attitudinal items on the severity of core ED symptoms within the past 28 days (we did not consider the six additional open-ended questions on the frequency of compensatory and binge behaviors). The items, which address Restraint, Eating Concern, Weight Concern, and Shape Concern, were rated on a 7-point scale (from 1, never, up to 7, every day). However, given the previously observed variance of the EDE-Q factor structure across gender groups (40, 41), we only calculated the global score (i.e., the mean across items, Cronbach’s α = 0.88) for the current analysis.

Body perception and body image were assessed using the FBeK [*Fragebogen zur Beurteilung des eigenen Körpers*, engl. Body Experience Questionnaire; (42)], which is a widely used questionnaire in Germany for assessing individuals’ subjective views of their own bodies (43). Its 52 dichotomous items (yes/no) assess Physical Attractiveness and Self-confidence (e.g., “I am attractive”; Cronbach’s α = 0.81), Accentuation of Physical Appearance (e.g., “The outer appearance says a lot about a person”; Cronbach’s α = 0.66), Insecurities and Concerns related to Appearance (e.g., “My appearance has already prevented me from connecting with others”; Cronbach’s α = 0.77), and Physical-Sexual Discomfort (e.g., “I am satisfied with my sexual sensations”; Cronbach’s α = 0.50). Gender-based percentile ranks of subscale means were provided for the present analysis.

#### 2.2.2. General psychopathology

The Symptom Checklist SCL-27-plus (44, 45) served as a validated brief measure of general psychopathology (46, 47). Twenty-seven items assess the presence of depressive (e.g., “melancholy”), vegetative (e.g., “heart palpitations”), agoraphobic (e.g., “becoming afraid of crowds”), and sociophobic symptoms (e.g., “feeling insecure when others look at me”) as well as pain (e.g., “chest pain”) over the last 2 weeks on a 5-point scale (from 1, *never*, to 5, *very often*). Further dichotomous questions address lifetime depressive symptoms and suicidality. However, as the proposed factorial validity received little empirical support (47), we only considered the overall score, the global severity index (GSI; Cronbach’s α = 0.90), for analysis.

The Beck Depression Inventory [BDI; (48)] was also included as a widely used self-report inventory to assess the presence and severity of depressive symptoms. The BDI contains 21 items, each scored on a 4-point scale, with sum scores ranging between 0 and 63 (Cronbach’s α = 0.87).

#### 2.2.3. Sociodemographic features

All individuals who were included in the study completed a standardized “life history” questionnaire with open and multiple-choice questions (category-coding in parentheses). The assessed sociodemographic features included: age in years, place of birth (Germany, others), marital status [single, married, divorced or separated, other (widowed or no response)], children (yes, no, no response), housing situation (living alone, living with others), currently in relationship (yes, no), secondary education [no degree/pupil, “Hauptschulabschluss” (lowest secondary graduation certificate, requiring 9 years of education), “Realschulabschluss” (intermediate secondary graduation certificate, requiring 10 years of education), “Fachhochschulreife” (advanced secondary graduation certificate, serving as technical college entrance qualification, requiring 11 years of education), “Abitur” (highest secondary graduation certificate required for university admission, usually received after 13 years of education)], occupational situation (full-time, part-time, not working), in retirement (yes, no), and upbringing conditions (with both parents, with single parent, with step-parents, other). Additional questions queried past mental disorders of mother and father (yes, no, no response), age at ED onset [adolescence (age 20 and younger), adulthood (age 21–35), middle age (age 36–46)], weight status at ED onset (underweight, normal weight, overweight), experience of physical and/or sexual abuse (yes, no), previous suicide attempts (yes, no), and prior psychological treatment (none, once, more than once). We did not consider further open-ended and rating questions regarding eating and weight control behaviors due to their overlap with standardized questionnaires.

### 2.3. Data aggregation and analysis

Overall scores for the EDE-Q, BDI, and SCL-27+, and subscale scores for the FBeK were aggregated according to each questionnaire’s specifications. Patterns among the 17 sociodemographic features listed above (not including gender) were identified using latent class analysis (LCA). LCA is a person-centered approach that creates distinct subgroups based on response patterns across multiple dimensions (35). Unlike cluster analysis, the grouping of individuals in LCA is model-based, meaning that the number of latent classes is determined by how well *k* number of classes fit the data. Moreover, LCA is probabilistic, meaning that LCA estimates the probability that an individual belongs to *k* number of classes. While latent classes are characterized descriptively, the estimated probabilities can be used to identify associations between patient features (e.g., gender and psychopathology) and class membership statistically. We capitalized on this feature to identify associations between patient gender, psychopathology, and patterns in sociodemographic dimensions.

The LCA was performed in three steps. First, we used the coded 17 indicators (see above) to construct latent class mixture models of increasing complexity, from *k* = 1 up to *k* = 10 classes, specifying multinomial and Gaussian response distributions for categorical and continuous variables (i.e., age), respectively. We then fitted the models repeatedly to the data using maximum likelihood estimation to ensure the stability of fit indices. Finally, we selected the best fitting model based on the obtained fit indices. Following recommendations for samples with *N* < 300 (35), we considered the two most frequently used measures of model fit for model selection, the Bayesian Information Criterion (BIC) and the Akaike Information Criterion (AIC), with lower values indicating better model fit.

Following the LCA, we conducted two sets of comparisons. First, we compared between men and women with EDs globally while controlling for effects of diagnosis group, using questionnaire scores and probabilities of latent class (LC) assignment as dependent variables, to answer whether men and women with EDs differ in sociodemographic patterns and clinical features overall. Here, we used “Type-II” analysis of variance (ANOVA), which estimates gender and diagnosis main effects without adjusting for potentially biased interaction variance resulting from the unequal distribution of specific diagnoses within gender groups (49, 50). Second, we compared questionnaire scores between sociodemographic LCs to determine how sociodemographic patterns relate to ED and general psychopathology. We ran these comparisons separately for men and women (i.e., within-gender), using one-way ANOVA, as LC assignment and gender were not independent (see Results below).

Descriptive results are reported as means, relative frequencies, and standard deviations (SDs). The significance level for all analyses was set at *p* ≤ 0.05. We applied Bonferroni correction for multiple comparisons on a per-questionnaire basis, and when analyzing class probabilities. We screened for univariate and multivariate outliers among questionnaire responses based on interquartile range (1st quartile – 3 × interquartile range, 3rd quartile +3 × interquartile range) and, respectively, Mahalanobis distance (for FBeK only) with *p* < 0.001 (51). There were seven multivariate outliers among the four FBeK subscales, which were removed for the FBeK’s analyses. Patients admitted before and after the onset of pandemic-related restrictions did not differ in psychopathology or sociodemographic characteristics, and we therefore did not consider this variable further. Effect sizes for ANOVAs are reported as partial *η^2^.* Case–control matching and one-way ANOVA was conducted using SPSS Statistics Version 28 for Windows (52). Type-II ANOVAs were calculated using R package car 3.1.1 (53). LCA was conducted using functions *mix* and *fit* in R package depmixS4 1.5.0 (54). The R version used was 4.2.2 (55).

## 3. Results

### 3.1. Sociodemographic latent classes

Table 1 summarizes the sample’s overall and class-dependent sociodemographic data. The information criteria of the fitted models of the 17 sociodemographic indicators suggested 4- and 5-class solutions (see Table 2): While the AIC favored the 5-class model, the 4-class model obtained the lowest BIC. Although BIC and AIC tend to perform similar in modestly-sized samples (56), we ultimately decided in favor of the more parsimonious BIC solution given that it yielded rather equally sized classes compared to the 5-class AIC solution (35).

**Table 1:** Patient sociodemographic features.

Variables	LC 1	LC 2	LC 3	LC 4	Total
*n*	67	38	58	45	208
Sex					
Male	27	17	39	21	104
Female	40	21	19	24	104
ED group					
AN	20	6	11	17	54
BN	10	3	5	4	22
BED	31	25	39	23	118
EDNOS	6	4	3	1	14
Variables included in LCA					
Age (M ± SD)	22.8 ± 3.3	46.7 ± 9.9	37.4 ± 10.2	16.4 ± 1.9	29.9 ± 0.9
Birthplace					
Germany	65	38	56	42	201
Other	2	0	2	3	7
Secondary education					
No degree/pupil	0	0	0	43	43
Lowest	11	6	7	0	24
Intermediate	17	12	22	2	53
Advanced	9	6	12	0	27
Highest	30	14	17	0	61
Occupation					
Not working	31	7	14	44	96
Working part-time	11	10	10	1	32
Working full-time	25	21	34	0	80
Retired					
Yes	6	7	18	6	37
No	61	31	40	39	171
Housing situation					
Living alone	10	2	51	0	63
Living with others	57	36	7	45	145
Marital status					
Single	58	2	53	39	152
Married	9	32	0	1	42
Divorced or separated	0	3	5	0	8
Other	0	1	0	5	6
Currently in relationship					
Yes	30	36	5	2	73
No	37	2	53	43	135
Children*					
Yes	0	28	5	2	35
No	22	9	22	4	57
No response	45	1	31	39	116
Upbringing conditions					
Both parents	48	30	44	27	149
Single parent	15	2	5	11	33
Step-parent	3	4	7	5	19
Other	1	2	2	2	7
Father with mental illness					
Yes	33	28	28	17	106
No	34	9	18	25	86
No response	0	1	12	3	16
Mother with mental illness					
Yes	41	25	27	18	111
No	26	9	19	23	77
No response	0	4	12	4	20
History of abuse					
Yes	19	19	17	4	59
No	48	19	41	41	149
Suicide attempts					
Yes	13	6	7	4	30
No	54	32	51	41	178
Age at ED onset					
Adolescence	59	23	37	45	164
Young adulthood	8	10	18	0	36
Middle adulthood	0	5	3	0	8
Weight at ED onset					
Underweight	7	3	10	4	24
Normal weight	32	14	22	25	93
Overweight	28	21	26	16	91
Prev. treatments					
None	18	6	14	24	62
Once	26	13	15	9	63
More than once	23	19	29	12	83

*Patients were queried for the number of their children; “no response” was coded if patients skipped the question, and we only coded “no” if so explicitly stated. LC, latent classes derived from latent class analysis (LCA); AN, Anorexia Nervosa; BN, Bulimia Nervosa; BED, Binge-Eating Disorder; EDNOS, Eating Disorder Not Otherwise Specified. LCA included all sociodemographic variables except for gender and eating disorder groups.

**Table 2:** Information criteria of fitted LCA models.

Classes	df	Log-likelihood	AIC	BIC
1	32	−3447.7	6959.4	7066.2
2	65	−3205.8	6541.6	6758.6
3	98	−3119.5	6435.0	6762.1
4	131	−3015.8	6293.6	**6730.8**
5	164	−2968.9	**6265.8**	6813.2
6	197	−2942.5	6279.0	6936.5
7	230	−2919.2	6298.5	7066.1
8	263	−2869.2	6264.3	7142.1
9	296	−2851.7	6295.4	7283.3
10	329	−2823.8	6305.7	7403.7

Lower AIC and BIC values indicate better model fit (lowest values in bold). df, degrees of freedom; AIC, Akaike information criterion; BIC, Bayesian information criterion.

We interpreted the 4-class solution as identifying differently aged patient populations in different social situations. One class (LC4) included, on average, adolescents still attending school, living with their parents or others, not being in a relationship, with a majority presenting for the first time for ED treatment. Another class (LC1) included young adults, highly educated, unmarried but partially engaged in romantic relations, and mostly living in some form of social arrangement (e.g., with parents, with peers, or with partners). A majority reported previous experience with one or more ED treatments and being of normal weight or overweight at ED onset. The two remaining classes (LC2 and LC3) separated within patients in middle age. One class (LC2) included married and committed patients with children, a majority with an active romantic relationship, having parents with a history of mental disorders, and (proportionally) the highest rate of experiencing abuse. In contrast, the other class (LC3) included single adults, who were living alone, were not engaged in a relationship, with few having children, and a majority working full-time. This class also contained the majority of cases with multiple previous treatment attempts.

### 3.2. Between-gender comparisons in latent class assignment and psychopathology

Results and descriptive statistics for the global comparison between men and women with EDs are summarized in Tables 3, 4, respectively. Gender had a significant effect on probability of assignment to LC 3 (“single-childfree-working”), with men having a 2.1 times higher risk to belong to the single-childfree-working class than women (0.38 vs. 0.18). No other class probabilities varied as a function of gender. Diagnosis did not affect LC assignment, meaning that diagnosis groups were distributed similarly across the classes.

**Table 3:** Results of type-II ANOVA between-gender comparisons in latent class assignment and psychopathology.

Variable	df error	Sex/gender (df = 1)			ED group (df = 3)			Interaction (df = 3)		
		*F*	*p*	*η_p_* ^2^	*F*	*p*	*η_p_* ^2^	*F*	*p*	*η_p_* ^2^
p of LCA										
LC1*	200	4.27	0.160	0.02	2.02	0.450	0.03	0.13	1	0.00
LC2*	200	0.52	1	0.00	1.27	1	0.02	0.76	1	0.01
LC3*	200	11.48	0.003	0.05	1.47	0.897	0.02	0.35	1	0.01
LC4*	200	0.31	1	0.00	1.86	0.550	0.03	0.82	1	0.01
BMI	200	13.61	<0.001	0.06	172.8	<0.001	0.72	1.02	0.386	0.02
EDE-Q	189	1.40	0.238	0.01	3.34	0.020	0.05	3.72	0.012	0.06
FBeK										
Phys. Attr.*	182	1.21	1	0.01	1.75	0.632	0.03	0.13	1	0.00
Accent.*	182	2.40	0.491	0.01	6.17	0.002	0.09	2.01	0.459	0.03
Insecur.*	182	0.80	1	0.00	0.94	1	0.02	1.43	0.942	0.02
Discom.*	182	4.63	0.131	0.02	3.29	0.088	0.05	0.32	1	0.01
SCL-27+ GSI	190	6.36	0.012	0.03	2.41	0.068	0.04	1.54	0.205	0.02
BDI	192	0.62	0.431	0.00	3.62	0.014	0.05	1.96	0.121	0.03

*p*-values for variables marked with an asterisk (*) are Bonferroni-adjusted. Phys. Attr., Physical Attractiveness and Self-confidence; Accent., Accentuation of Physical Appearance; Insecur., Insecurities and Concerns related to Appearance; Discom., Physical-Sexual Discomfort.

**Table 4:** Descriptive statistics for between-gender comparisons (mean ± SD).

Variable	Gender	AN	BN	BED	EDNOS	Total
*n* (m/w)		27/27	11/11	59/59	7/7	104/104
p(LC1)	Men	0.32 ± 0.45	0.37 ± 0.48	0.20 ± 0.38	0.34 ± 0.46	0.26 ± 0.42
	Women	0.40 ± 0.47	0.54 ± 0.52	0.32 ± 0.44	0.57 ± 0.53	0.38 ± 0.46
p(LC2)	Men	0.11 ± 0.31	0.19 ± 0.40	0.19 ± 0.39	0.14 ± 0.38	0.16 ± 0.37
	Women	0.12 ± 0.32	0.09 ± 0.30	0.24 ± 0.42	0.43 ± 0.53	0.20 ± 0.40
p(LC3)	Men	0.31 ± 0.45	0.36 ± 0.49	0.41 ± 0.48	0.38 ± 0.47	0.38 ± 0.47
	Women	0.11 ± 0.28	0.09 ± 0.30	0.25 ± 0.41	0.01 ± 0.01	0.18 ± 0.36
p(LC4)	Men	0.26 ± 0.45	0.08 ± 0.26	0.20 ± 0.40	0.14 ± 0.38	0.20 ± 0.40
	Women	0.37 ± 0.49	0.27 ± 0.47	0.19 ± 0.39	0.00 ± 0.00	0.23 ± 0.42
BMI	Men	17.30 ± 2.52	26.90 ± 7.16	47.22 ± 7.95	31.24 ± 16.20	36.23 ± 15.22
	Women	16.21 ± 1.65	21.67 ± 2.76	41.92 ± 9.96	29.15 ± 7.48	32.24 ± 13.93
EDE-Q	Men	4.60 ± 1.13	5.45 ± 1.35	4.06 ± 0.92	3.68 ± 1.42	4.32 ± 1.16
	Women	4.62 ± 1.16	4.40 ± 1.85	4.40 ± 1.05	5.15 ± 0.95	4.51 ± 1.16
FBeK						
Phys. Attr.	Men	6.92 ± 10.36	3.30 ± 5.60	4.39 ± 4.53	4.29 ± 4.50	4.93 ± 6.66
	Women	5.19 ± 6.93	1.83 ± 1.60	3.87 ± 5.77	2.67 ± 1.97	4.04 ± 5.83
Accent.	Men	82.48 ± 20.13	78.30 ± 27.21	61.37 ± 24.80	83.57 ± 16.07	70.25 ± 25.22
	Women	75.81 ± 23.71	96.33 ± 4.23	71.55 ± 24.68	81.00 ± 20.62	74.96 ± 23.95
Insecur.	Men	87.68 ± 8.53	88.40 ± 19.34	79.83 ± 24.38	84.14 ± 23.22	83.08 ± 20.84
	Women	80.96 ± 17.94	92.83 ± 6.34	85.73 ± 17.38	92.33 ± 8.85	85.23 ± 16.86
Discom.	Men	91.00 ± 10.35	94.60 ± 10.38	82.33 ± 20.51	87.14 ± 18.99	86.22 ± 17.80
	Women	92.81 ± 11.00	96.83 ± 6.82	88.93 ± 15.24	93.33 ± 12.23	90.83 ± 13.61
BDI	Men	27.81 ± 9.72	30.55 ± 14.62	21.79 ± 10.88	25.00 ± 10.23	24.54 ± 11.35
	Women	29.33 ± 8.63	21.11 ± 14.37	24.36 ± 9.28	28.33 ± 9.29	25.65 ± 9.85
SCL27+ GSI	Men	1.71 ± 0.63	2.05 ± 0.95	1.39 ± 0.68	1.76 ± 0.45	1.57 ± 0.72
	Women	1.88 ± 0.53	1.75 ± 0.96	1.77 ± 0.69	1.92 ± 0.20	1.81 ± 0.65

AN, Anorexia Nervosa; BN, Bulimia Nervosa; BED, Binge-Eating Disorder; EDNOS, Eating Disorder Not Otherwise Specified; Phys. Attr., Physical Attractiveness and Self-confidence; Accent., Accentuation of Physical Appearance; Insecur., Insecurities and Concerns related to Appearance; Discom., Physical-Sexual Discomfort.

ED-related psychopathology, as assessed by the EDE-Q and FBeK scores, did not differ between men and women. We obtained a Gender × ED group interaction on the EDE-Q global scores, descriptively driven by higher EDE-Q scores in men with BN compared to women with BN, and lower EDE-Q scores in men with EDNOS compared to women with EDNOS. However, given the potential bias in estimating interaction effects with unequally-sized subgroups (49, 50), we caution against further interpretation.

Comparisons in general psychopathology showed that men had lower overall symptom scores in the SCL-27+ than women, suggesting that men with an ED diagnosis presented with less severe general burden. BDI depression scores were comparable between men and women.

### 3.3. Within-gender comparisons of latent classes

**Figure 1:**
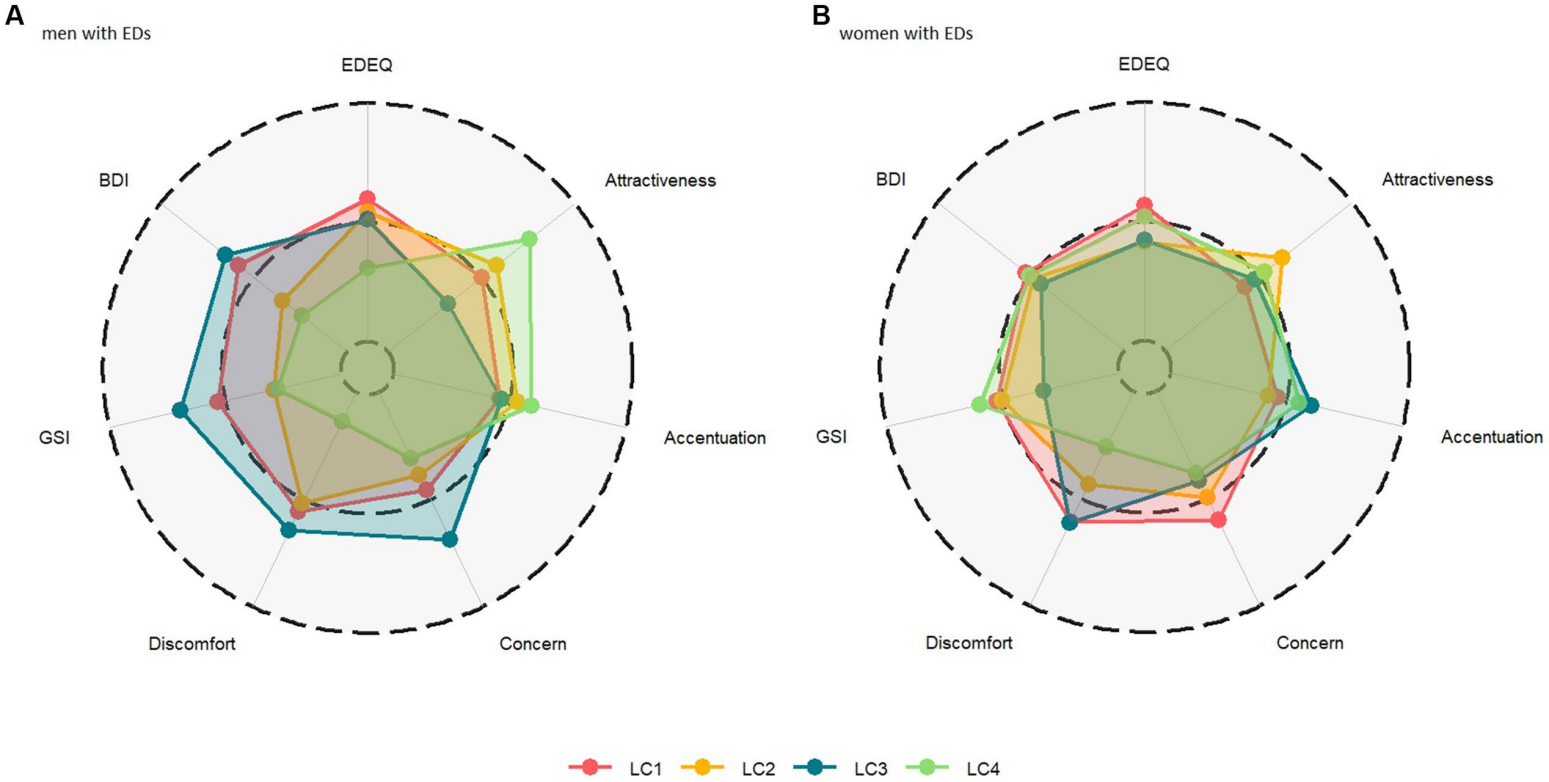
Radar chart of z-standardized questionnaire scores means for **(A)** men with EDs (left panel) and **(B)** women with EDs (right panel) as a function of latent class (LC) assignment. The dashed middle line represents the subsample mean, with the outer and inner rings indicating +1 SD and −1 SD of the mean, respectively.

Finally, results and descriptive statistics for the comparisons between men and women assigned to different sociodemographic LCs are presented in Table 5 and Figure 1, respectively. EDE-Q scores did not differ between men assigned to different classes. However, FBeK Physical/Sexual Discomfort scores varied between men assigned to different classes, with men in LC3 presenting with the highest sexual discomfort scores that differed significantly from men’s scores in LC4, *p* = 0.002 (men in LC3 also presented with the lowest attractiveness scores, though the overall class differences were not significant following Bonferroni correction). Moreover, SCL27+ global burden scores (GSI) differed among men with EDs, with men assigned to LC3 (“single-childfree-working”) presenting with the highest scores that differed significantly from men’s scores in LC2 and LC4, *p*s < 0.03. Men in LC3 also presented with the highest BDI scores, which differed significantly from men in LC4, *p* = 0.02. In contrast, among women, EDE-Q, FBeK, SCL-27+, and BDI scores did not differ between different LCs.

**Table 5:** ANOVA results of univariate within-gender comparisons of latent classes.

Variable	Gender	df error	*F*	*p*	*η_p_^2^*
EDE-Q	Women	96	0.56	0.642	0.02
	Men	96	1.38	0.253	0.04
FBeK					
Phys. Attr.*	Women	90	0.70	1.00	0.02
	Men	92	3.55	0.068	0.10
Accent.*	Women	90	0.56	1.00	0.02
	Men	92	0.37	1.00	0.01
Insecur.*	Women	90	1.05	1.00	03
	Men	92	3.15	0.116	0.09
Discom.*	Women	90	2.68	0.204	0.08
	Men	92	4.99	0.012	0.14
SCL-27+ GSI	Women	96	1.10	0.354	0.03
	Men	94	4.55	0.005	0.13
BDI	Women	96	0.11	0.955	0.00
	Men	96	4.05	0.009	0.11

*p*-values for variables marked with an asterisk (*) are Bonferroni-adjusted. df_factor_ = 3. Phys. Attr., Physical Attractiveness and Self-confidence; Accent., Accentuation of Physical Appearance; Insecur., Insecurities and Concerns related to Appearance; Discom., Physical-Sexual Discomfort.

## 4. Discussion

In this study we compared clinical and sociodemographic features between matched samples of men and women with EDs who presented for inpatient treatment. We found that patients could be grouped into four distinct latent classes based on their sociodemographic features, and that men with EDs had a significantly higher risk to belong to a “single-childfree-working” class than women. Moreover, and although overall men with EDs presented with similar ED-related psychopathology and even lower general psychopathology scores than women with EDs, within-gender comparisons between latent classes revealed different results. Here, we found that single-childfree-working men presented with significantly higher general psychopathology compared to men in other classes, whereas the assigned sociodemographic class had no impact on scores in women with EDs.

The finding that men with EDs, when matched in sample size and diagnoses, are more likely single and childfree, and that these men specifically present with higher psychopathology, may suggest that the absence of a “traditional” model of social support (i.e., a romantic partnership and a family with children) and disordered eating could be particularly interconnected in men. Those who deviate from social expectations to follow a particular life course, including finding a partner and having children, may face social pressure, judgment, and stigmatization (57). This can lead to feelings of inadequacy, a sense of not belonging, or isolation, which may aggravate existing ED-related psychopathology (58). A previous longitudinal study found that, although adolescent men and women share several risk factors for the development of disordered eating, a lack of social support directly predicted disordered eating only in men and also increased the effect of depressive symptoms on eating-related psychopathology (32). These findings are consistent with the sociodemographic patterns observed in our sample and could support the hypothesis that disordered eating can arise in men as an attempt to cope with social isolation concerns. If future research with even larger samples further supports this hypothesis, clinicians and therapists may want to specifically focus on issues of interpersonal relations and social isolation in disordered eating screening and treatment for men.

Moreover, healthcare professionals should be aware that having fewer intimate relations or social ties could also be a consequence of disordered eating especially in men, thus contributing towards worse overall mental health. Qualitative studies show that boys and young men face challenges in recognizing their own symptoms and seeking treatment, and may delay help-seeking due to feared or experienced social stigma associated with EDs being a “women’s disease” (16). Likewise, ED-related stigmatization, both internalized (leading to feelings of shame, self-doubt, and low self-esteem) and socially imposed (associated with discrimination and rejection), may create barriers to forming and maintaining intimate connections in men with EDs due to fears of being labeled, judged, or perceived as weak (59), or increase sexual risk behaviors to avoid partner rejection (60). Addressing stigma is therefore crucial for promoting understanding, empathy, and healthy bonding. This requires raising awareness and challenging negative stereotypes and beliefs in both the general public as well as among healthcare professionals.

### 4.1. Limitations

Although our data suggest that the psychopathology of men with EDs is linked to the absence of a “traditional” model of social support, we cannot yet determine the specific nature of what this might entail. For example, we have no data on the presence (or absence) of relationships between friends and peers which could substitute for the absence of romantic relationships. We have also neither assessed aspects like personal values, goals, feelings of loneliness, nor the ability to cope with adversity (i.e., resilience), which will be crucial for developing and testing the assumed processes that could explain our findings.

On a more general level, we must also note that our study does not include longitudinal data. EDs can often change over the course of life, and we are unaware whether and how these changes relate to associations of psychopathology and sociodemographic features. Our analyses were also limited to a sample of inpatients sharing various admission prerequisites, such as meeting certain diagnostic criteria, a certain level of psychological stability, a completed preadmission interview (for data completion), and sufficient motivation for therapy. Our sample did not include acute cases of malnutrition, or cases considered merely at risk for disordered eating, which may limit generalizations toward broader patient and general population samples. Additionally, there are several indications that diagnostic criteria and ED assessment tools (such as the EDE-Q) may be suboptimal in capturing ED psychopathology in men (41, 61), which may have artificially homogenized our patient sample.

We were also unable to examine diagnosis-specific patterns due to the limited size of the individual diagnostic group, suggesting the need for larger-scale investigations. Relatedly, and although diagnostic group and sociodemographic clusters were unrelated, we must caution against over-generalizing the latent class solution, given our sample is not representative of all patients with EDs. A majority of the sample included patients with BED, and we cannot exclude that observed associations between gender and psychopathology within specific clusters could change in differently composed or larger samples.

Finally, on a conceptual note, we must acknowledge that our data provide no clear distinction between effects of patient sex (i.e., male and female status typically assigned at birth) vs. patient gender (i.e., a person’s deeply felt, inherent sense of being, for example, a man or woman). Even if we assume that the observed effects may be attributed to the influence of gender and gender-associated sociocultural norms (14), we cannot make that distinction based on the collected data. Other studies also highlight the importance of sexual orientations for body image concerns and dissatisfaction (62), which were not assessed here but should be examined in addition to sex and gender in future investigations. Moreover, we would still need to identify the relevant aspects at work (gender-associated concepts of the self, personality characteristics, coping styles, etc.). Clearly, further research is needed with regard to such aspects as well.

## 5. Conclusion

There are currently only few studies examining gender differences between men and women with EDs. This study explored the differences in sociodemographic features and psychopathology in men and women with EDs. One key difference showed that men with ED have a 2.1 times higher risk than women to belong to a single-childfree-working subgroup based on their sociodemographic features, and that this group in particular shows higher general symptom burden. Further studies on gender differences are needed to investigate specific gender effects, develop new diagnostic tools, improve access to treatment, and possibly implement gender-adapted treatments.

## Data availability statement

The raw data supporting the conclusions of this article will be made available by the authors, without undue reservation.

## Ethics statement

The studies involving human participants were reviewed and approved by Ethics Committee of the Ruhr-University Bochum’s Medical Faculty at Campus East-Westphalia. Written informed consent for participation was not required for this study in accordance with the national legislation and the institutional requirements.

## Author contributions

The present work was performed by PT in fulfillment of the requirements for obtaining the degree “Dr. med.” PT, GH, and GP contributed to conception and design of the study. PT, GH, and KB organized the database. GH and PT performed the statistical analysis. PT wrote the first draft of the manuscript. GH and GP wrote sections of the manuscript. All authors contributed to the article and approved the submitted version.

## Conflict of interest

The authors declare that the research was conducted in the absence of any commercial or financial relationships that could be construed as a potential conflict of interest.

## Publisher’s note

All claims expressed in this article are solely those of the authors and do not necessarily represent those of their affiliated organizations, or those of the publisher, the editors and the reviewers. Any product that may be evaluated in this article, or claim that may be made by its manufacturer, is not guaranteed or endorsed by the publisher.

## Footnotes

^1^Please note that we use both terms, sex and gender, in conjunction as most studies rarely distinguish between patient sex (i.e., male and female status typically assigned at birth) and patient gender (i.e., a person’s deeply felt, inherent sense of being, for example, a man or woman).
